# Modelling viscous boundary layer dissipation effects in liquid surrounding individual solid nano and micro-particles in an ultrasonic field

**DOI:** 10.1038/s41598-019-40665-9

**Published:** 2019-03-20

**Authors:** Derek Michael Forrester, Jinrui Huang, Valerie J. Pinfield

**Affiliations:** 1 Loughborough University, Chemical Engineering, Loughborough, LE11 3TU United Kingdom; 2 Present Address: Qinetiq Group plc., Cody Technology Park, Ively Road, Farnborough, Hampshire, GU14 0LX United Kingdom; 3 Present Address: Department of Mechanical Engineering, Imperial College, London, SW7 2AZ United Kingdom

## Abstract

Upon application of ultrasonic waves to a suspension of solid particles in liquid, multiple scattering occurs at the particle/liquid interfaces leading to attenuation. It was recently shown through experimental verification that multiple scattering theory must include shear wave influences at the boundary between the liquid and solid particles in a nanofluid when the concentration of the scatterers is even as low as a few percent by volume. Herein, we consider silica spheres of 50–450 nm diameter in the long-wavelength regime to elucidate the form of the shear decay fields at the liquid/solid interface for individual particles. This is important because the overlap of these fields ultimately leads to the conversion of a compressional wave to shear waves and back into the compressional wave, the effect originating due to the density contrast between the particle and the liquid. Therefore, we examine in detail the velocity, vorticity and viscous dissipation in the shear wave field and around the silica spheres using finite element modelling, giving clarity to the viscous boundary effects. We also compare the numerical modelling to semi-analytical results.

## Introduction

In the analysis of solid particles in liquids, the size, density, etc. of the scatterer is perhaps best evaluated through ultrasonic spectroscopy when the concentration is high enough to make the sample impenetrable by light^1–3^. This can happen at very low concentrations, rendering traditional light scattering techniques inadequate^4^. Ultrasonic methods can be employed successfully for characterisation of a sample or product^5–9^. However, there remains uncertainty over the accuracy of using this modality because many existing models for evaluation of the spectra overestimate the attenuation found experimentally, particularly as the concentration of particles increases^1,10^.

It is believed that the reason for this is that consideration of the short-range decay fields produced by the compressional wave at the particle interfaces are commonly neglected^10–13^. As concentration increases these decay fields overlap with neighbouring particles, leading to a reduction in the dissipation of energy, and a recombination effect with the compressional wave - meaning less attenuation. Thus, we are interested in investigating the shear^1^ field around a solid spherical scatterer, such as silica, in a viscous liquid, such as water^1^. This is important for understanding the nano- and micro-particle effects in industrial processes^14^, biological systems^15^, and environmental pollution using ultrasound^16^ (to name but a few areas).

The characterisation of particulate systems and the application of ultrasonic manipulation to influence particle dynamics (e.g. for separation and sorting^17,18^), relies on a thorough understanding of the shear effects occurring in clusters or ensembles of particles in a viscous liquid and their dependence on parameters such as frequency and particle size^19,20^. In order to do this, a detailed description of the amplitude and extent of the decay fields around silica clusters needs to be determined, and this is founded in the single-particle system investigated here. We have recently reported simulations of the thermal decay fields around liquid particles in a liquid medium, which lead to thermal interactions between particles in a cluster^21^. Here, we report simulations of the shear wave fields around a single (solid) silica particle with diameter 50 nm to 450 nm in water, and the influence of particle size and frequency on the wave amplitude, wavelength, and viscous dissipation in the region of the particle. These simulations act as a building block for the investigation of the interactions between particles mediated by the shear fields in concentrated or aggregated particle systems.

## Details of Finite Element Simulations

**Figure 1:**
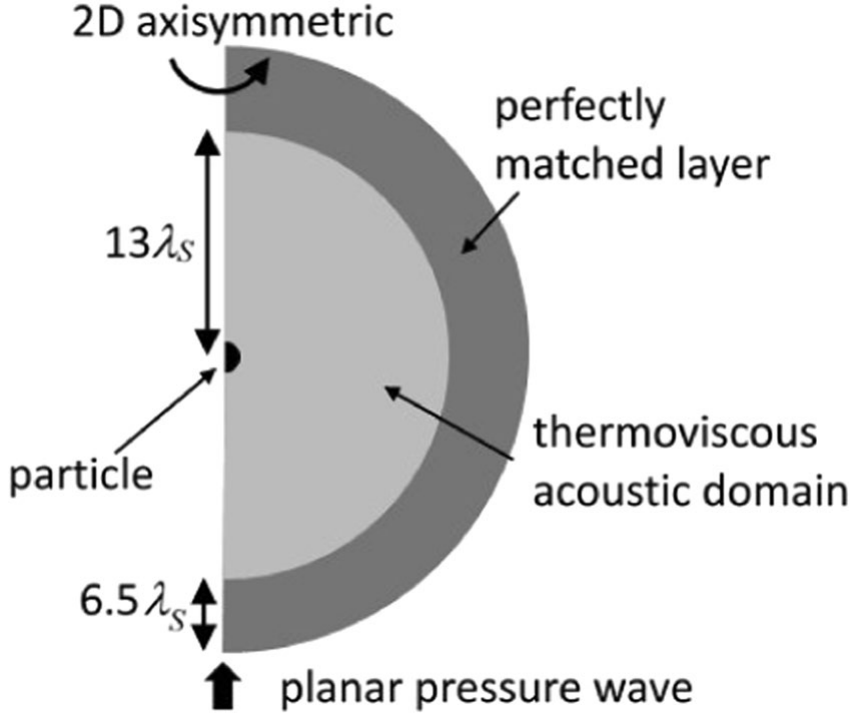
The simulations model an ultrasonic plane wave propagating in the z-direction applied to a spherical silica particle in water. The system is 2D axi-symmetric.

In this work we use COMSOL Multiphysics (version 5.3)^22^, a commercial finite-element based software package, to model the thermo-viscous effects around a single spherical silica particle (*SiO*_2_) in water, in a planar acoustic field, using the set-up shown in Fig. 1. The thermoviscous acoustics (TVA) frequency domain module is used throughout this work to calculate the temperature, pressure, and velocity variations near the walls of the silica where viscous and thermal losses are important. The equations defined by the TVA interface are the linearised Navier-Stokes equations in quiescent background conditions solving the continuity, momentum, and energy equations. The model is 2D axisymmetric and the TVA region is surrounded by a perfectly matched layer (see Fig. 1) to avoid reflections from the boundary. The simulations performed in this work were carried out using a workstation with two Intel Xeon CPU E5-2630v3, 2.40 GHz processors and 256 GB of DDR4 RAM. In this section we present the simulation system.

### The thermoviscous acoustic equations

The frequency-domain TVA model is based upon the linearised Navier-Stokes equations for a harmonic field of angular frequency ***ω*** of the form exp (*i****ω****t*). The Thermoviscous Acoustic Model node is used to define the model inputs such as the background temperature (298.15 K) and the pressure (we use the default value of 1 atm), together with the equilibrium material properties from the material model (see section below). The equations of the system are as below (note **I** is the identity matrix)):

1 $$i\omega {\rho }_{0}{\bf{u}}=\nabla \cdot (-p{\bf{I}}+{\mu }_{S}[\nabla {\bf{u}}+{(\nabla {\bf{u}})}^{{\bf{T}}}]-[\frac{2}{3}{\mu }_{{\bf{S}}}-{\mu }_{{\bf{B}}}][\nabla \cdot {\bf{u}}]{\bf{I}}),$$

2 $$i\omega \rho +{\rho }_{0}\nabla \cdot {\bf{u}}=0,$$

and

3 $$i\omega {\rho }_{0}{C}_{p}T=\nabla \cdot (\kappa \nabla T)+i\omega p{T}_{0}{\varepsilon }_{0},$$

where ***ω*** is angular frequency, *μ*_*S*_ is shear viscosity, *μ*_*B*_ is bulk viscosity, *C*_*p*_ is heat capacity at constant pressure, ***κ*** is thermal conductivity, and ***ε***_0_ is the volume thermal expansion coefficient at constant pressure. The time-harmonic perturbations in pressure, velocity, temperature and fluid density (*p*, **u**, *T*, and ***ρ*** respectively) are written without subscripts, these being superimposed upon their steady state values (indicated by subscript 0); thus ***ρ***_0_ is steady state density. The equations (1–3) are solved in the thermo-visco-acoustic liquid domain, together with the density variation ***ρ*** = ***ρ***_0_(***β***_*T*_*p* − ***ε***_0_*T*), where ***β***_*T*_ is isothermal compressibility. The incident perturbing field is defined as a planar background pressure field propagating in the z-direction.

4 $$p=|{p}_{b}|{e}^{-i{k}_{b}\hat{{\bf{z}}}},$$

with the normalised wave direction vector $\hat{{\bf{z}}}$ and |*p*_*b*_| = 0.1 MPa. The simulation framework is linear and therefore all perturbations scale with the specified pressure amplitude. The background velocity in response to the pressure perturbation is,

5 $${{\bf{u}}}_{b}=\frac{\omega ({\beta }_{T}{p}_{b}-{\varepsilon }_{0}{T}_{b})}{{k}_{b}}\hat{{\bf{z}}}$$

and the background temperature is

6 $${T}_{b}=\frac{i{\boldsymbol{\omega }}{{\boldsymbol{\varepsilon }}}_{0}{T}_{0}}{i{\boldsymbol{\omega }}{{\boldsymbol{\rho }}}_{0}{C}_{p}+{\boldsymbol{\kappa }}{k}_{b}^{2}}{p}_{b}$$

Herein, the background acoustic field wavenumber takes the form,

7 $${k}_{b}=\frac{{\boldsymbol{\omega }}}{c}{[1+\frac{i\omega {b}_{tv}}{{\rho }_{0}{c}^{2}}]}^{-1/2}$$

with parameter

8 $${b}_{tv}=\frac{4}{3}{\mu }_{S}+{\mu }_{B}+\frac{(\gamma -1){\boldsymbol{\kappa }}}{{C}_{p}}.$$

where ***γ*** is the ratio of specific heat capacities and *c* is the adiabatic wave speed for compressional waves. These background field equations are taken from the COMSOL documentation, and are derived from the linearised time-harmonic field equations (1–3) by expressing them in Helmholtz form using a velocity potential, resulting in wavenumber *k* for each mode. The three wave modes (compressional/acoustic, thermal and shear) are denoted by subscripts *c*, *T* and *S* respectively; the wavenumber for the incident compressional background wave *k*_*b*_ = *k*_*c*_. The scattered field is superimposed on these background harmonic fields to obtain the full perturbation field values for each quantity. The solid mechanics module is used to define the properties of the silica particle which is isotropic and linearly elastic. The initial displacement and structural velocity fields are set to zero. Coupling between the solid particle and the TVA domain is carried out in the Multiphysics Node of the software. The coupling gives the relationship between solid displacement and total fluid velocity **u**_*t*,*fluid*_ = *i****ω*u**_*solid*_ at the particle boundary, where **u**_*t*,*fluid*_ is the total fluid velocity including the background field and *u*_*solid*_ is the displacement of the (boundary of the) silica sphere. Thus displacement is continuous at the boundary; stress is also continuous at the boundary.

### The model structure

We are interested in the shear and thermal fields around the particle, which have typical length scales that are orders of magnitude smaller than the compressional (acoustic) wavelength in the systems under investigation. These fields form thermal and viscous boundary layers, which have been studied since the early analysis of Stokes^23^ and others, and later extensively by Schlichting^24^. The system must therefore be defined in terms of the wavelengths of the shear or thermal fields. Here, since we study a solid particle where scattering is dominated by visco-inertial effects, it is the shear wavelength that is used to define the characteristic scale of the system.

The shear and thermal decay fields have wavelengths,

9 $${\lambda }_{S}\,=\,2\pi \sqrt{\frac{{\mu }_{S}}{\pi {\rho }_{0}\,f}},$$

and

10 $${\lambda }_{T}=2\pi \sqrt{\frac{\kappa }{\pi {\rho }_{0}\,f{C}_{p}}},$$

respectively, where *f* is frequency. Axial symmetry is set along the *z* axis at *R* = 0 (the cylindrical radial coordinate). The initial values of perturbed pressure, velocity, and temperature are all set to zero. The problem is set around the particle in a region stretching 13 shear wavelengths (see Eqn. 9) from the particle centre in all directions. The particle sits in an off-set position with its centre at (*R*, *z*) = (0, 13***λ***_*S*_). The axial boundaries are defined as within the ranges [*R*_*min*_, *R*_*max*_] = [0, 19.5***λ***_*S*_] and [*z*_*min*_, *z*_*max*_] = [−6.5***λ***_*S*_, 32.5***λ***_*S*_], including the PML. The particle radius is denoted *a*.

The typical wave speed for the PML is set here to 1497 m/s (speed in water at 25 °C) and all temperatures are adjusted to 298.15 K. For stability the discretisation for pressure is set one element order less than for velocity and temperature. Linear element order is set for pressure, and quadratic order for velocity and temperature. The order for temperature and velocity are kept the same due to our requirement to model thermal and viscous boundary layers. The dependent variables are pressure *p*, velocity field *u*, and the temperature variation *T* (all taking complex values).

### The mesh and perfectly matched layer

**Figure 2:**
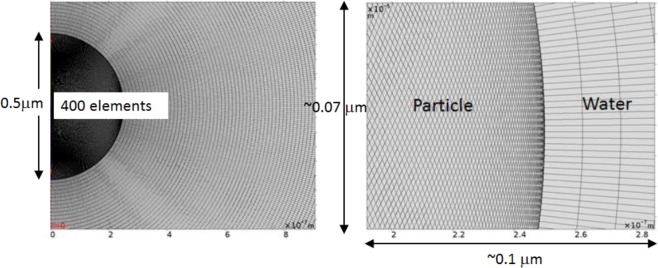
A typical mesh, shown here for a 500 nm diameter silica particle, in a water domain. On the z-axis (vertical) there are 1100 elements outside the particle (water area) each of 12.5 nm and 400 elements on the z-axis of the region of the particle each of 1.25 nm. The model is axisymmetric. The silica particle is analysed for varying diameters.

The mesh is “mapped” according to two distributions emanating from the line of axial symmetry inside the particle and outside it (within the TVA domain). Inside the particle the symmetry axis is split into 400 elements of equal length 1.25 nm. Likewise the symmetry axis either side of the particle in the TVA domain is split into 1100 elements each of 12.49 nm length (which means approximately 86 elements fit in one shear wavelength). The result is a radial cross meshing with tight enough sizing so that radial spread has limited effect. The meshing inside the TVA and solid mechanics domains can be seen in Fig. 2. Further refinement of the mesh does not improve the simulations^21^.

The perfectly matched layer (PML) surrounds the TVA domain as shown in Fig. 1 and is designed to absorb all outgoing wave energy. This removes an issue of impedance mismatch at the boundary with the TVA. A predefined “fine” COMSOL triangular mesh is defined for the PML. In the PML conditions we set the type to spherical with a polynomial stretching type. The PML is designed to apply a complex coordinate stretching in 1–3 directions, determined from how it connects to the physical domain. The stretching is a function of the wavelength associated with the frequency of the applied ultrasonic wave. The width of the PML is chosen to be 6.5 shear wavelengths (***λ***_*S*_). The complex displacement for stretching in a single direction is Δ**x** = ***λ***_*w*_*f*_*p*_(*ξ*) − Δ_*w*_*ξ*, where ***λ***_*w*_ is the wavelength (*c*_*water*_/*f*) and Δ_*w*_ = 6.5***λ***_*S*_. The *ξ* is a dimensionless coordinate that varies from 0 to 1 over the PML. The polynomial stretching function *f*_*p*_(*ξ*) = *sξ*^*p*^(1 − *i*) where *p* is a curvature parameter (set to 1 here) and *s* is a scaling function (also set to 1). As the shear and thermal waves have almost completely decayed after the chosen propagation distance to the boundary with the PML a curvature factor of 1 is more than sufficient to ensure satisfactory mesh resolution within the PML. The pressure amplitude is found to be zero inside the PML.

### Material properties

The physical properties used in the modelling can be found in Table 1. All other values are determined from these properties; isothermal compressibility, for example, is determined from the (adiabatic) sound speed and is *β*_*T*_ = *γ*/*ρ*_0_*c*^2^ = 4.51 × 10^−10^ Pa^−1^). The calculated Poisson’s ratio and shear wavespeed have also been checked for self-consistency. These properties define the shear wavenumber which in turn determines the size of the simulated domain since we are interested in the shear wave decay.

**Table 1:** Physical parameters of silica and water (at 25 °C) entered as material values in the finite element modelling.

	Silica	Water
Specific heat capacity, *C*_*p*_ (J/kg.K)	—	4179
Shear viscosity, *μ* (Pa.s)	—	0.000891
Ratio of specific heats, *γ*	—	1.007
Density, *ρ* (kg/m^3^)	2200	997
Bulk modulus, *K*, (GPa)	37.16	—
Shear modulus, *G*, (GPa)	30.9	—
Thermal conductivity, *κ*, (W/m.K)	—	0.595
Speed of sound, *c*, (m/s)	5968	1497
Bulk viscosity, *μ*_*B*_, (mPa.s)	—	2.47
Coeff. of thermal expansion, _0 ε_, (1/K)	—	0.00021

## Results

Simulations were conducted for silica particles of diameter 50–450 nm in water with a planar ultrasonic field propagating in the *z* direction. Selected frequencies in the range 15–400 MHz were used, corresponding to a range of dimensionless shear wavenumber 0.09 < *k*_*S*_*a* < 4 where *a* is the particle radius. Hence we cover the “‘resonance”’ condition where *k*_*S*_*a* ≈ 1 such that the shear wavelength is comparable to the particle diameter; according to theory, shear field effects are most significant at this condition. Although our ultimate aim is to investigate particle interactions through the shear field, here we present the results of the simulations for a single particle and show the effect of particle size and frequency on shear wave field effects to establish the single particle effects before studying particle interactions.

**Figure 3:**
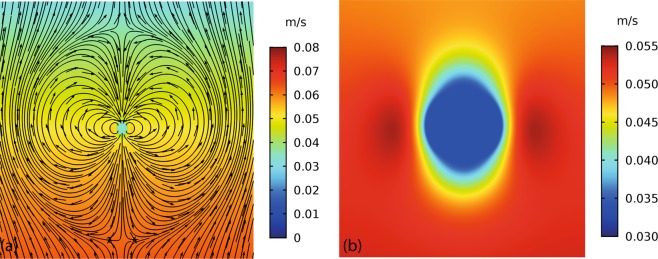
Velocity profiles around a 450 nm diameter particle at 15 MHz. (**a**) The dipolar form of total acoustic velocity field is highlighted through streamlines around a particle. (**b**) The *z*-component of the sum of the scattered and background velocity.

**Figure 4:**
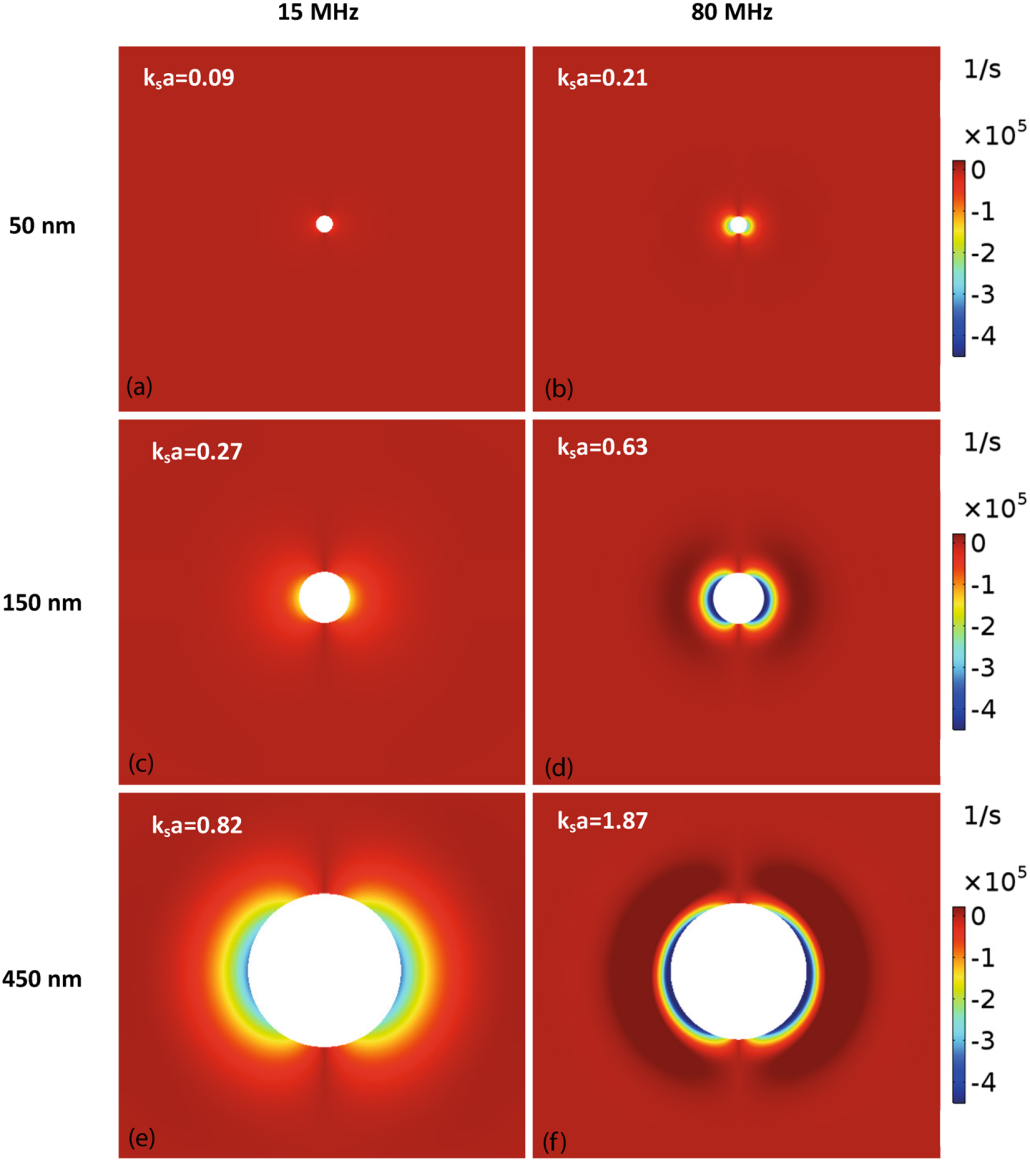
Images of the magnitude of the azimuthal component of vorticity for particles of different sizes at frequencies of 15 MHz ((**a**,**c**) and (**e**)) and 80 MHz ((**b**,**d**) and (**f**)). The values of the real part of the dimensionless shear wavenumber *k*_*s*_*a*, which determines the magnitude of the shear wave field are shown on the figures.

Figure 3(a) shows the typical form of the total velocity field around a particle, illustrating the predominantly dipole nature of the field due to the density difference between particle and liquid which causes relative motion between the particle and the bulk liquid (at some distance from the particle). The circulation of the liquid in the region around the particle is clear. The axial component of the velocity (*u*_*z*_) is shown in Fig. 3(b), demonstrating that the particle’s greater inertia causes it to have a lower velocity in the propagation direction than that of the liquid at some distance from the particle. These velocity fields can be used to determine the vorticity

11 $${\rm{\Omega }}=\nabla \times {\bf{u}}$$

which relates only to the (vector) shear wave field (vorticity is zero for an acoustic wave field) and shows the magnitude of the circulating motion near the particle; only the azimuthal component of vorticity is non-zero in this symmetric system. The vorticity is shown in Fig. 4 for particles of different diameters at two different frequencies. The side lobes in these images show the region of most intense vorticity, nearest the particle surface. The decay of vorticity with distance from the particle surface is rapid; at higher frequency the decay distance is smaller as the shear wavelength decreases, and the shear field is confined to the region very close to the particle (compare, for example Fig. 4(e,f)). The decay distance is a smaller proportion of the particle radius as *k*_*s*_*a* increases and correspondingly *d*/***λ***_*S*_ also increases, where *d* is particle diameter. The images also indicate that the vorticity magnitude increases with increasing particle size.

**Figure 5:**
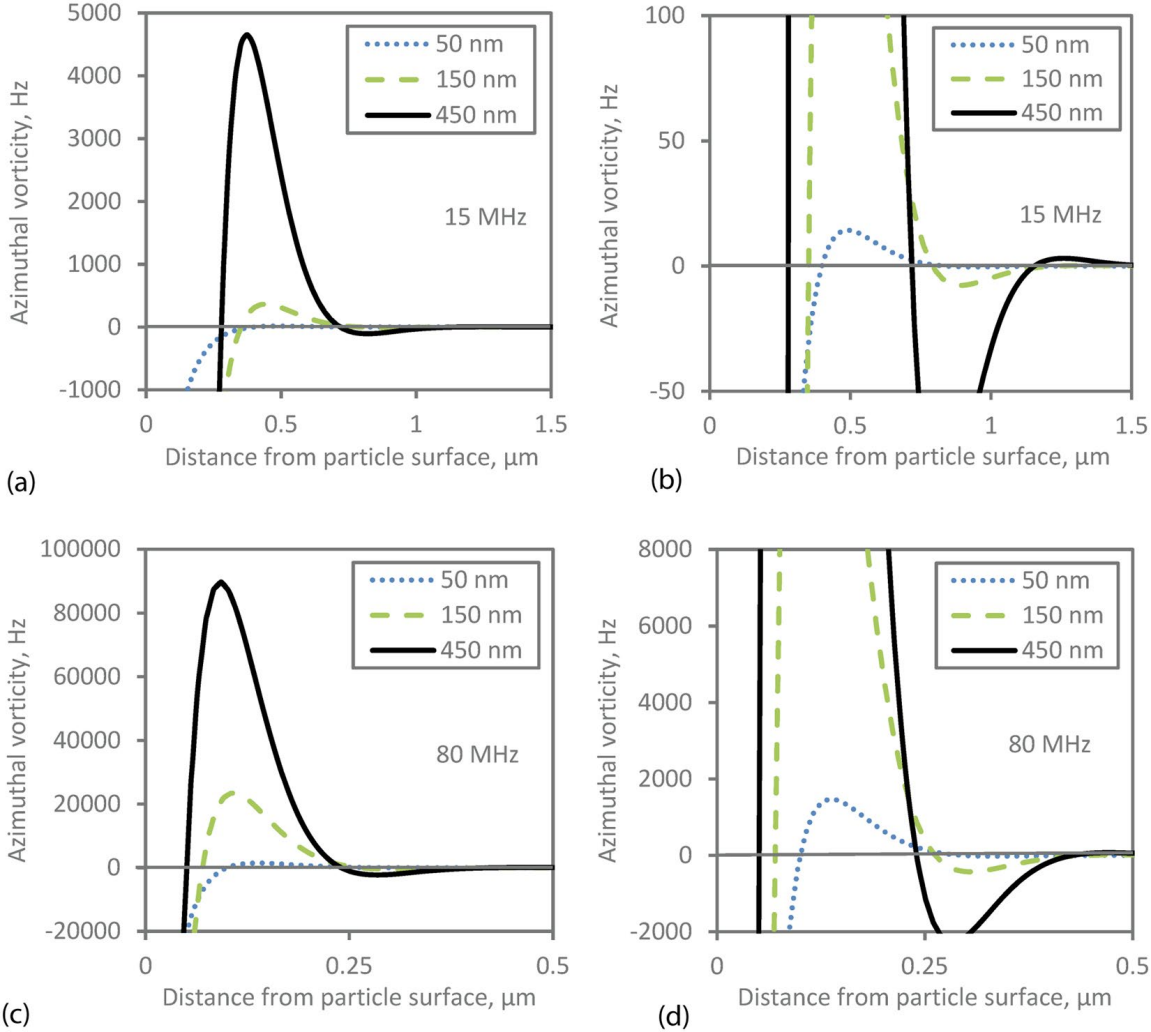
Azimuthal vorticity along the radial direction plotted from the particle surface (zero position is at the particle surface) for different particle sizes and frequencies of (**a**,**b**) 15 MHz and (**c**,**d**) 80 MHz. The plot is taken at a fixed z-position (*z* = 13*λ*_*S*_) - see section 2.2. The right hand plots (**b**) and (**d**) show an expanded version of the plots (**a**,**c**) respectively, so that the second turning point can be more clearly seen. The vorticity increases rapidly as the particle surface is approached but the finite value of vorticity at the surface is not shown here due to the axis scale which was chosen to make the oscillations visible.

To examine the vorticity in more detail, it is plotted in Fig. 5 as a function of distance from the particle surface in the radial direction at fixed *z* = 13***λ***_*S*_ (i.e. at the *z*- coordinate of the particle centre) for selected frequencies and particle sizes. Maxima and minima are observed in the vorticity, demonstrating its wave-like nature relating to the shear-wave field. Whilst the peak of vorticity varies to a great extent with particle size, the intervals between the nodes (zero crossings) are similar for each particle size at fixed frequency. These intervals are presented for particle sizes of 50 nm and 450 nm in Tables 2 and 3, and are compared with the value of half the shear wavelength *λ*_*S*_/2, which could be expected to match the intervals for a shear wave field.

**Table 2:** Intervals (a–c) between consecutive zero crossings of the azimuthal vorticity along the radial direction for a particle diameter of 50 nm and a frequency of 15–400 MHz.

Frequency/(MHz)	(a)/*μ*m	(b)/*μ*m	(c)/*μ*m	(*λs*/2)/*μ*m
15	0.442	0.437	0.427	0.433
80	0.194	0.189	0.189	0.187
150	0.143	0.138	0.138	0.137
400	0.085	0.085	0.082	0.084

(a) is between the first and second zero crossing, (b) between second and third zero crossing etc. The value of half the shear wavelength is shown for each frequency.

**Table 3:** Intervals (a–c) between consecutive zero crossings of the azimuthal vorticity along the radial direction for a particle diameter of 450 nm and a frequency of 15–400 MHz.

Frequency/(MHz)	(a)/*μ*m	(b)/*μ*m	(c)/*μ*m	(*λs*/2)/*μ*m
15	0.440	0.436	0.429	0.433
80	0.189	0.188	0.189	0.187
150	0.138	0.138	0.138	0.137
400	0.084	0.084	0.080	0.084

(a) is between the first and second zero crossing, (b) between second and third zero crossing etc. The value of half the shear wavelength is shown for each frequency.

The velocity field of the incident (compressional) wave (the “‘background field”’) is written as a scalar velocity potential ${\rm{\Phi }}$

12 $${{\bf{u}}}_{{\bf{b}}}=-\,\nabla {\rm{\Phi }}$$

and the shear wave velocity field is written as vector velocity potential ${\rm{\Psi }}$ which has only an azimuthal component (due to the spherical symmetry)

13 $${{\bf{u}}}_{{\bf{S}}}={\rm{\nabla }}\times {\rm{\Psi }}$$

Each of the wave potentials in the Rayleigh limit for this spherical problem can be expressed as a sum over partial wave orders. The dipole partial wave contribution (denoted by a subscript 1) to the incident field can be expressed as^25,26^

14 $${{\rm{\Phi }}}_{1}=3i{{\rm{\Phi }}}_{0}{j}_{1}({k}_{c}r){P}_{1}(cos{\boldsymbol{\theta }})$$

where **Φ**_0_ is the amplitude of the velocity potential of the incident planar acoustic wave, *j*_1_ is the spherical Bessel function, *P*_1_ is a Legendre polynomial, and ***θ*** is the polar angle. The scattered shear wave for this dipole partial wave is defined by the dipole part of the azimuthal component of the velocity potential ${\rm{\Psi }}$_1_ which takes the form

15 $${{\rm{\Psi }}}_{1}=3i{T}_{1}^{CS}{{\rm{\Phi }}}_{0}{h}_{1}({k}_{S}r){P}_{1}^{1}(cos{\boldsymbol{\theta }})$$

where ${T}_{1}^{CS}$ is the scattering coefficient of the shear wave (with incident compressional wave), *h*_1_ is the spherical Hankel function of the first kind and ${P}_{1}^{1}$ is an associated Legendre polynomial. It can therefore be shown analytically, using equations 11 and 15, that the azimuthal component of the vorticity for this dipole field is given by

16 $${{\rm{\Omega }}}_{\psi }=-\,3i{T}_{1}^{CS}{{\rm{\Phi }}}_{0}\frac{{k}_{S}^{2}{r}^{2}{h^{\prime\prime} }_{1}({k}_{S}r)+2{k}_{S}r{{h}^{^{\prime} }}_{1}({k}_{S}r)-2{h}_{1}({k}_{S}r)}{{r}^{2}}{P}_{1}^{1}(cos{\boldsymbol{\theta }})$$

The intervals between the nodes of this function can only be solved numerically, and are close to but not exactly equal to *λ*_*S*_/2. However, the close agreement here between FE simulations and the shear wavelength provides evidence that the shear field is being resolved correctly.

**Figure 6:**
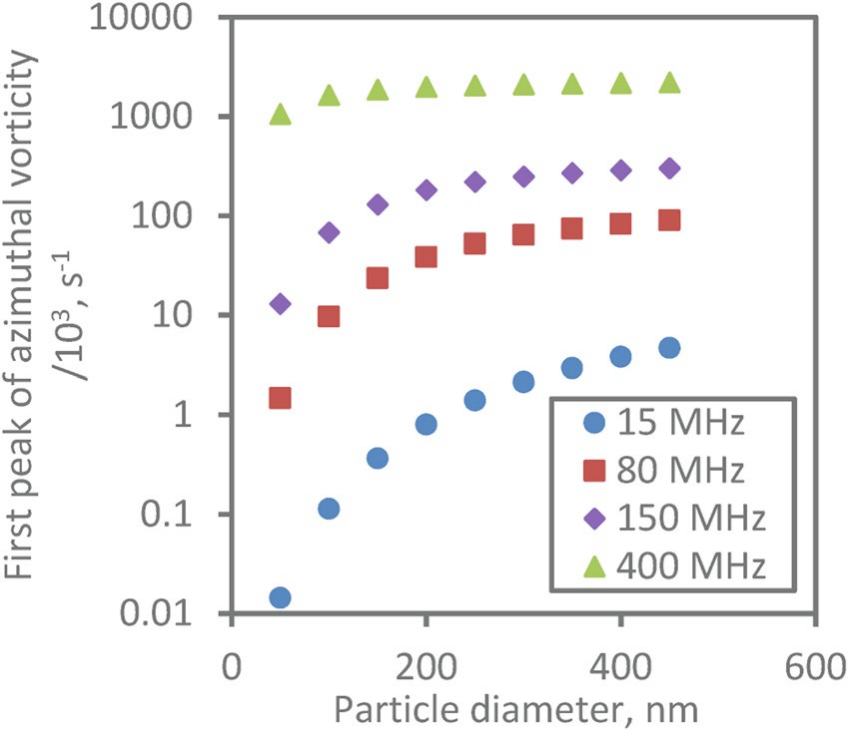
The magnitude of the first peak of the azimuthal component of vorticity as a function of particle diameter at a range of frequencies.

In Fig. 6 we show the effect of particle size and frequency on the magnitude of the shear wave field, by taking the amplitude of the first peak of the azimuthal vorticity when plotted against distance (e.g. as shown in Fig. 5). Here, we see that increases in both particle size and frequency lead to higher peak vorticity. The analytical vorticity expression for a dipole field (Eqn. 16) shows, however, that the vorticity does not scale simply with dimensionless shear wavenumber *k*_*s*_*a*, being also influenced by the compressional wavenumber *k*_*c*_*a* through the scattering coefficient. It is not therefore possible to reduce the dependence of peak vorticity to a simple scaling graphically or analytically; hence the particle size and frequency effects are considered separately.

**Figure 7:**
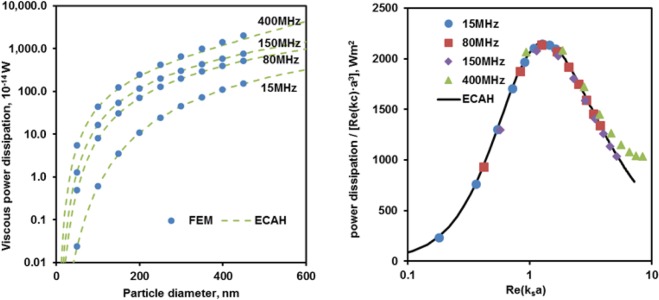
Viscous power dissipation due to the particle (i.e. with the dissipation in the bulk liquid subtracted) as a function of particle diameter at different frequencies. (**a**) shows the power dissipation compared with the prediction of Rayleigh partial wave scattering model (labelled ECAH) and (**b**) shows the scaled power dissipation as a function of dimensionless shear wavenumber *k*_*s*_*a* illustrating the peak effects around *k*_*s*_*a* ≈ 1. The ECAH data was calculated using a frequency of 15 MHz and range of particle sizes; results for higher frequencies are identical within the long wavelength region i.e. *k*_*c*_*a* ≪ 1 but deviate beyond that condition.

Having observed the vorticity around the particle relating to the shear wave produced by the dipole motion, we now examine the power dissipated due to the shearing motion. This power dissipation relates directly to the attenuation detected for an acoustic wave propagating through an ensemble of particles in a viscous liquid. Here, for a single particle, the dependence of the viscous power dissipation on particle size and frequency is shown in Fig. 7. The power dissipation is determined using the built-in COMSOL viscous power dissipation function, from which the viscous power dissipation in bulk water is subtracted to obtain the excess dissipation due to the presence of the particle; the method is presented in more detail by Forrester and Pinfield^21^. The resulting net power dissipation is compared with the analytical prediction of the Epstein-Carhart, Allegra-Hawley model^25,26^ in Fig. 7, showing that the modal Rayleigh partial wave model for the monopole and dipole modes only, is consistent with the finite element solution. The power dissipated in the shear wave field is higher for larger particles and for higher frequencies Fig. 7(a). However, by scaling the power dissipation it is possible to identify the contribution which varies solely with the dimensionless shear wavenumber Fig. 7(b). The net dissipated power^25^ is given by

17 $$P=-\,2\pi \frac{{p}_{b}^{2}}{\omega {k}_{c}\rho }{\boldsymbol{\Re }}({T}_{0}^{CC}+3{T}_{1}^{CC})$$

where ${T}_{\mathrm{0,1}}^{CC}$ denotes the scattering coefficient of the compressional wave mode for an incident compressional wave, for monopole and dipole wave orders 0 or 1 respectively; the wave amplitude (taken as unity in ref.^25^) is related to the pressure amplitude by

18 $${{\rm{\Phi }}}_{0}=\frac{i{p}_{b}}{\omega \rho }$$

Since the scattering coefficients take the form,

19 $${T}_{0,1}^{CC}={({k}_{c}a)}^{3}{F}_{0,1}({k}_{S}a)$$

in the long acoustic wavelength limit *k*_*c*_*a* ≪ 1, the power dissipation therefore scales as

20 $$P={k}_{c}{a}^{3}G({k}_{S}a)c{p}_{b}^{2}$$

where *F* and *G* are functions of *k*_*s*_*a*. Figure 7(b) therefore shows that the scaled viscous power dissipation varies with the dimensionless shear wavenumber, with a peak power dissipation at *Re*(*k*_*s*_*a*) ≈ 1. A factor of *c* (the sound speed in water) remains, however, and the power scaling is therefore not universal. This is consistent with the analytical results and experimental data which shows that the attenuation per wavelength for dilute suspensions varies with *k*_*s*_*a* and has a peak at this condition, representing a shear wave resonance condition. Here, simulations at different particle sizes and frequencies fall onto a single curve on the plot Fig. 7(b); some deviation occurs where the conditions are no longer in the long compressional wavelength condition *k*_*c*_*a* ≪ 1 for the largest particles and highest frequencies (*k*_*c*_*a* = 0.38 at 400 MHz for 400 nm diameter particles).

## Discussion

The viscous energy losses around a particle in an acoustic field are both particle size and frequency dependent as demonstrated by these simulations, but analytically, experimentally and by simulation it can be shown that the maximum losses occur at the condition *k*_*s*_*a* 1 in dilute systems. This corresponds to a shear wave resonance where the shear wavelength is comparable to the particle size. In suspensions in which particles are on average closer together than the viscous decay length, the shear wave field influences neighbouring particles, modifying the scattered acoustic field, resulting in a reduction in the energy lost in viscous dissipation^6^. This is similar to a Schlichting boundary layer becoming as large as the gap between two surfaces. The system must then be treated in a fully viscous framework rather than the field near the surfaces behaving independently of each other. In the case of dispersions of particles, shear wave interactions between particles will occur if the interparticle separation (between particle surfaces) is less than the shear wave length (or similarly viscous decay length or boundary layer thickness)

21 $$\langle s\rangle  < {\lambda }_{S}.$$

**Figure 8:**
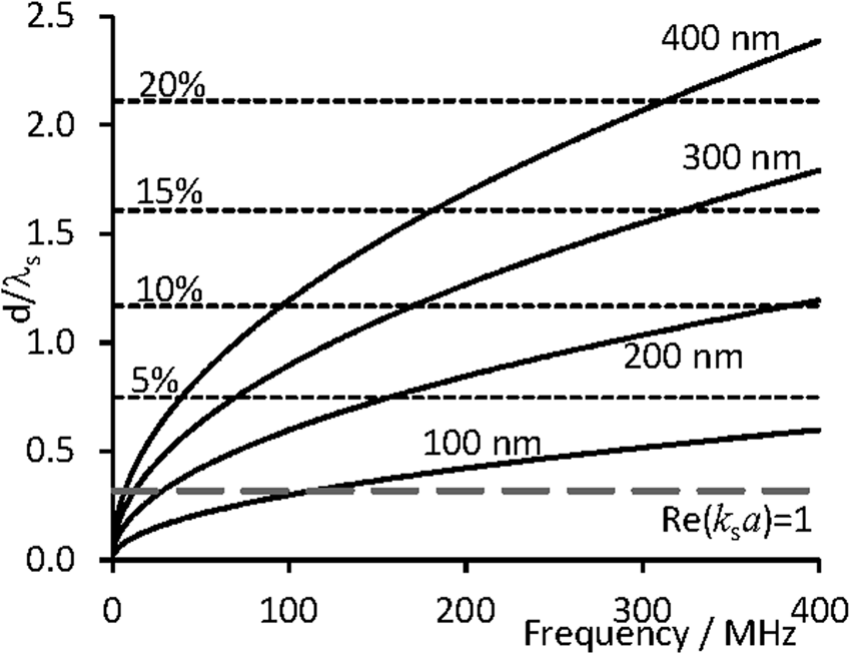
The ratio of particle diameter to shear wavelength at different sizes and frequencies. The resonance condition at which peak viscous dissipation losses occur (${\boldsymbol{\Re }}({k}_{s}a)=1$) is shown by the grey long-dashed line. The maximum value of $\frac{d}{{\lambda }_{S}}$ for which shear wave particle interactions are expected to occur are shown by the dotted lines for a range of concentrations.

Since the average distance between particle surfaces is estimated as

22 $$\langle s\rangle =d[{(\frac{{{\boldsymbol{\varphi }}}_{rcp}}{{\boldsymbol{\varphi }}})}^{\mathrm{1/3}}-1],$$

where *d* = 2*a* is the diameter of the particles, ***ϕ***_*rcp*_ is the random close packed volume fraction (≈0.64, for hard spheres) and ***ϕ*** is volume fraction of particles, the condition specifies an upper limit on the ratio *d*/***λ***_*S*_ at which shear interactions exist between particles. Values of *d*/***λ***_*S*_ below this limit have viscous boundary layers which extend beyond the average distance between particle surfaces, and therefore the shear wave fields will interact and modify the overall acoustic field. A reduction in viscous power dissipation ensues; experimental evidence for this phenomenon is reported in reference.^1^. These conditions are shown on Fig. 8 for a range of concentrations; the resonance condition ${\boldsymbol{\Re }}({k}_{s}a)=1$ is also highlighted. Large ratios *d*/*λ*_*S*_ imply larger separations at the same volume fraction, and hence a lower degree of shear interactions. For the particle sizes studied here, concentrations even as low as 5% by volume can lead to shear interactions as other particles occupy the viscous boundary layer created around each particle.

## Conclusions

Herein, we have demonstrated the finite element analysis of a series of single solid particles of differing sizes in water as they interact with an ultrasonic plane wave. Solid particles in liquids, as concentration increases, are primarily affected by shear-fields (rather than thermal), and so we have focused upon the vorticity, velocities, and viscous power dissipation. The simulations demonstrated that the thickness of the viscous boundary layer (and the corresponding shear wavelength) decreased at higher frequencies, whilst the magnitude of the peak vorticity increased, consistent with expectations for a viscous boundary layer. An increase in particle size also led to higher vorticity and viscous power dissipation for the single particle. The losses due to viscous dissipation scale with the dimensionless shear wavenumber, and peak at the resonance condition ${\boldsymbol{\Re }}({k}_{s}a)=1$, consistent with findings experimentally and analytically^1^. Having simulated the shear wave field around a single particle, and explored its particle size and frequency dependence, the interactions between neighbouring particles can now be investigated using the same framework. In particular we aim to establish the change in viscous dissipation losses which relate to attenuation in suspensions of concentrated or aggregated particles due to particle occupancy within the viscous boundary layers, an effect which has been observed experimentally^1^.
